# Asymptomatic malaria reservoirs are the last challenge in the elimination in Cambodia

**DOI:** 10.1186/s12936-025-05343-4

**Published:** 2025-04-05

**Authors:** Dyna Doum, David J. McIver, Ingrid Chen, Vanney Keo, Siv Sovannaroth, Dysoley Lek, Joanne M. Cunningham, Diane D. Lovin, Nicholas W. Daniel, Molly Quan, Elodie Vajda, Allison Tatarsky, Neil F. Lobo

**Affiliations:** 1 Malaria Elimination Initiative, Institute for Global Health Sciences, University of California, San Francisco, USA; 2 Health Forefront Organization, Phnom Penh, Cambodia; 3 National Center for Parasitology, Entomology and Malaria Control, Phnom Penh, Cambodia; 4 University of Notre Dame, Notre Dame, IN USA; 5 Swiss Tropical and Public Health Institute, Basel, Switzerland; 6 University of Basel, Petersplatz 1, CH-2003 Basel, Switzerland

**Keywords:** Malaria, Malaria elimination, Asymptomatic, Prevalence, QPCR, *Plasmodium*

## Abstract

**Background:**

Malaria, a mosquito-borne disease, is a serious public health issue globally and a leading cause of morbidity and mortality in many developing countries worldwide. Cambodia is in the last stages of malaria elimination and aims to eliminate all species of human malaria by 2025. Despite tremendous progress, eliminating malaria in Cambodia has proven to be challenging due to pockets of residual transmission in high-risk populations sustained by untreated asymptomatic malaria reservoirs. Understanding the extent of asymptomatic malaria reservoirs in ‘last-mile’ communities such as those in Mondulkiri and Kampong Speu, is vital for an effective malaria elimination strategy.

**Methods:**

Malaria cross-sectional surveys were conducted in high-risk populations (forest dwellers, forest goers and forest rangers) at three different time points (T0, T1, T2) from October 2022 to February 2023, overlapping the rainy, malaria transmission season and into the dry season. Blood samples (n = 6350) collected on filter paper from participants from all target groups were screened for *Plasmodium* species using qPCR.

**Results:**

All qPCR-diagnosed cases were asymptomatic, indicating an untreated parasite reservoir. In Mondulkiri, the prevalence of *Plasmodium falciparum* was 0.63% at T0, increasing to 0.81% at T1, and decreasing to 0.18% at T2. *Plasmodium vivax* decreased from 4.80% at T0 to 1.97% at T1 and 1.65% at T2. In Kampong Speu, overall prevalence was 7.06% at T0, declining to 5.19% at T1 and 4.59% at T2. *Plasmodium falciparum* prevalence was 0.30% at T0, decreasing to 0.09% at T1 and rising slightly to 0.10% at T2. The forest goers showed a prevalence increase to 1.95% at T1 and decrease to 1.46% by T2, while forest dwellers decreased to 3.25% at T1 and further to 3.13% at T2. Passively reported malaria case showed that 1.09% of cases in Mondulkiri and 0.21% of cases in Kampong Speu were rapid diagnostic test (RDT) positive.

**Conclusion:**

Evidence generated during this study point to the continued presence of an untreated asymptomatic reservoir in high-risk populations. Targeted epidemiological and/or vector-based intervention strategies tailored to specific risk groups may enable a reduction of this sustaining reservoir of parasites, thereby leading to eliminating malaria in Cambodia.

## Background

Cambodia has historically reported high malaria transmission since the 1950s [1]. The country has a tropical environment that is home to a wide range of mosquitoes, including *Anopheles* [2]. People living or working in or near forested regions are at high risk for malaria infection [3]. Compared to neighboring countries like Vietnam, Thailand, and Laos, as well as the WHO Western Pacific Region as a whole, Cambodia continued to have the highest malaria burden [4]. However, in recent years, significant progress has been made towards the elimination of malaria. Between 2010 and 2020, the number of malaria cases decreased significantly from 106,228 to 9,771 cases, representing a reduction of 90.8% [1, 5], and Cambodia now represents 13.4% of the total number of cases in the Southeast Asia region [1, 5]. Being in the last stages of malaria elimination [4], Cambodia aims to eliminate all species of human malaria by 2025 [6, 7].

As Cambodia is in the malaria elimination stage, a large number of RDT-based screenings are conducted by Village Malaria Workers (VMWs) to treat asymptomatic cases when malaria foci are identified. These RDT screenings run parallel with Active Fever Screening (AFS), Target Drug Administration (TDA), and Intermittent Preventive Treatment for forest goers (IPTf). AFS consists of weekly fever door-to-door screening using RDTs, while TDA consists of administering a complete 3-day artemisinin-based combination therapy (ACT) regimen within the malaria focus for people who plan to work in forested areas within the following month for two consecutive months at the beginning of focus response activities. This active push towards malaria elimination is supervised by VMWs, with RDT data contributing to overall Malaria Information System (MIS) numbers. Data from the Health Centre (HC) records consist of mostly symptomatic cases with patients reporting to the HC for treatment when symptomatic.

Despite this progress, eliminating malaria in Cambodia has proven to be challenging for several reasons including the presence of asymptomatic malaria reservoirs [8]. Asymptomatic malaria, often referred to as the "silent threat" [9], is represented by individuals who are infected with malaria but do not exhibit any symptoms and do not seek treatment. These patients might also be subpatent (are negative with RDTs) and, therefore, escape detection and treatment. These people may harbour *Plasmodium* infections that can infect vectors for long periods, contributing to the spread of the disease in the community [10]. Identifying and treating these asymptomatic carriers are crucial in the final push toward eliminating malaria in Cambodia. However, this is difficult since diagnosis and treatment of malaria in Cambodia is health facility based and dependent on symptomatic cases in conjunction with a positive malaria test—typically RDTs or microscopy.

The presence of asymptomatic malaria reservoirs makes it difficult to achieve complete eradication of malaria, as they can continually transmit the disease without being detected and eliminated through treatment. To date, there remains a reservoir of submicroscopic malaria infections in the country [11]. A contributing factor may also be incomplete treatment or resistance to anti-malarial drugs, which may not eliminate the parasite from an infected person [12]. This study aimed to characterize the asymptomatic reservoir of parasites in three targeted high-risk populations (forest goers, forest dwellers, and forest rangers) from the high-transmission rainy season into the dry season in malaria hot spot communities of Mondulkiri and Kampong Speu province (s).

## Methods

### Study area and population

**Fig. 1:**
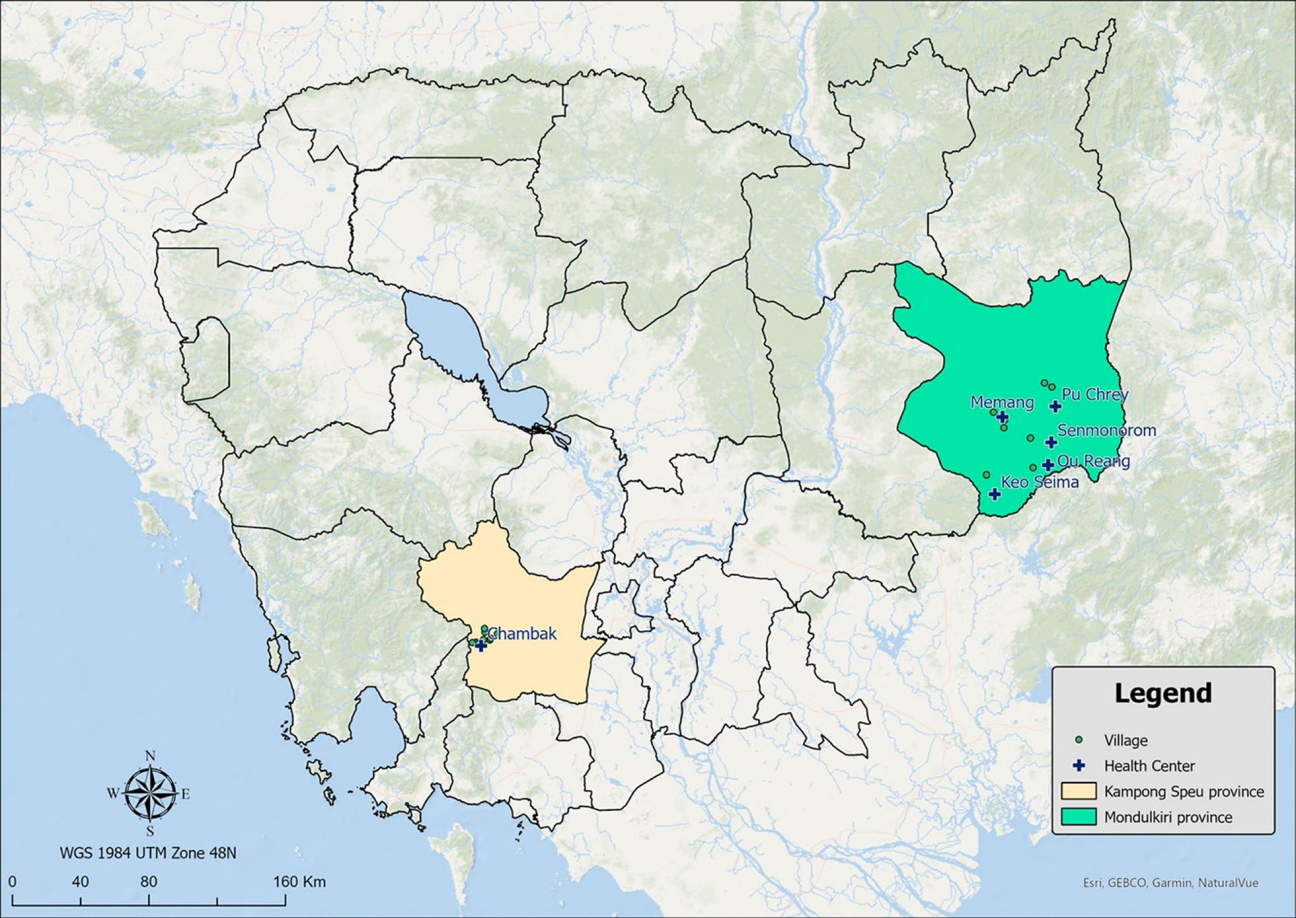
Study areas for data collection in Mondulkiri and Kampong Speu provinces, Cambodia

This study was conducted in Mondulkiri and Kampong Speu Provinces, Cambodia (Fig. 1), where malaria peak case rates usually occur from August to January during the rainy season. Kampong Speu and Mondulkiri are two of the five priority provinces for *Plasmodium falciparum* elimination within the National Center for Parasitology, Entomology, and Malaria Control (CNM) national strategy. These two provinces contribute nearly 40% of all *P. falciparum*/mixed cases and 36% of total cases reported in the country in 2021. Sampling villages were chosen based on ongoing local malaria passive case reporting (ongoing vector-based transmission) and consultations with local health authorities, with the villages with the highest reported number of cases included in the study.

Active malaria transmission foci in Cambodia are most often related to forested areas, with forest exposure being documented as a risk factor for malaria [13, 14]. Individuals at high risk of malaria infection that were targeted for blood sample collection within the villages included forest goers, forest dwellers, and forest rangers [15–20]. (1) Forest goers were defined as individuals who lived at least one kilometre from the forest and who travelled to the forest regularly, with forest activities often comprised of seasonal farming, hunting, or foraging for mushrooms, vegetables, and resin, as well as seasonal forest workers who migrate for gem mining, logging, and work in plantations; (2) Forest dwellers lived either in the forest permanently or lived within 1 km from the edge of the forest, largely surviving on subsistence farming. Forest dwellers often live in a permanent, traditional house in a non-forest village during part of the year, and in a more open, temporary structure in the farm or forest during planting and harvesting seasons; (3) Forest rangers are typically men working for government or wildlife and conservation agencies to protect the forest and areas near international borders, staying in ranger stations or outdoor hammocks up to 16 nights per month when they are on patrol.

This study is part of a larger, parent study called Project BITE (Bite Interruption Toward Elimination) which aimed to evaluate the entomological protective efficacy and effectiveness, cost, and acceptability of bite prevention tools among high-risk populations in Cambodia [21]. The parent study had specific inclusion and exclusion criteria, and participation required meeting all criteria. Participants had to be residents of villages targeted by the implementation feasibility study for BITE tool distribution, meet the definition of one of the three target populations, and be willing and available to conduct follow-up discussions. Participants also needed to be 3 years of age or above and speak Khmer and/or Bunong. Finally, they were required to provide informed consent if aged 18 years or above, or have their guardian provide informed consent if below 18 years of age.

### Sample size calculation

For the sample size estimate, the parent study [21], utilized an epidemiological outcome to allow for comparison of data between each timepoint. Paired proportions were compared using a baseline malaria prevalence of 6.7% [8] and an estimated reduction in malaria infections of 30% for users of the forest pack distributed in the parent study, as compared to non-users. This calculation indicated that 2100 participants were required for the cohort to detect differences in malaria epidemiology between each timepoint with 80% power and 95% confidence.

### Cross-sectional surveys

An implementation feasibility study was conducted among forest-exposed individuals, including cross-sectional surveys at three points in time. Study staff visited both households and ranger stations to screen for eligibility, obtain informed consent, administer questionnaires, and perform finger prick blood collections. Informed consent was obtained from all participants, including parental consent for any participant younger than 18 years. A survey questionnaire was administered to all participants, capturing individual and household-level demographic information, use of vector control tools, history of malaria, and treatment-seeking behaviour. The survey questionnaire proposed at T0 was indeed repeated at T1 and T2 to ensure consistency and track changes over time.

Baseline data was collected in October 2022 (Timepoint 0; T0), followed by additional surveys in December 2022 (T1), when additional participants were recruited, to compensate for loss to follow up (LTFU) participants, and in February 2023 (T2). Forest packs with mosquito bite prevention tools were delivered to participants beginning after T0. The results presented here focus on baseline risks of malaria infection amongst new participants recruited at T0 and T1, focusing on demographics including how open their living structures are, previous history of malaria infection, time spent in the forest, and malaria prevention tools in use prior to study initiation.

Blood samples were collected via finger prick from participants on filter paper. A total of 6,350 samples were obtained during the three survey time points (T0, T1, T2) between October 2022 and February 2023. If a participant had a fever (> 37 °C), a malaria RDT (SD Bioline Malaria Ag P.F/Pv) was administered. Positive cases were referred to the closest health facility for treatment. Filter paper (Whatman 3MM) was pre-cut into squares, with each card holding five blood spots of approximately 25 µl each. Samples were labeled with barcodes or ID numbers, allowed to dry, and then the card was closed.

Filter paper samples were labelled, appropriately stored and transported from the field in a Ziploc bag then placed in a stock card filter paper box with desiccant and humidity indicator card and stored at 4 ˚C within one week, and at − 20˚C within one month. DBS were regularly transported to the district or regional offices for refrigerated storage prior to bulk transport and shipment to designated processing laboratories.

### Sample processing and *Plasmodium* detection by qPCR

Blood samples were collected (~ 50 µl blood via finger prick) from each participant for a total of four dried blood spots (DBSs) per individual on filter paper (Whatman Filter Paper #3). All blood samples were screened for *Plasmodium* species infection using QuantaBio Perfecta qPCR ToughMix, Low Rox and appropriate probes [22–24].

### Data analysis

Descriptive statistics were used in the data analysis to look at the prevalence of *P. falciparum* and *Plasmodium vivax* malaria at three different time points (T0, T1, and T2) for both symptomatic and asymptomatic infections. The number of people included at each time point was T0 = 2111, T1 = 2192, and T2 = 2047. Malaria prevalence was also assessed based on specific target groups, including forest goers, dwellers, and rangers. Additionally, the incidence of symptomatic and asymptomatic malaria (*P. falciparum* and *P. vivax*) was analyzed at the three time points, utilizing a completed cohort of 1,011 individuals from the target groups across all time points. Furthermore, the national MIS-based incidence reported during the study period was investigated.

### Health facility and village malaria worker data

Passive malaria incidence data is generated by VMWs and the HC. VMWs are trained to diagnose malaria using RDT and treat uncomplicated malaria cases [25]. Symptomatic villagers visit VMWs or HCs for RDT screening and malaria treatment if positive. VMWs or HCs record all cases that are tested, confirmed, and treated, electronically entered into the Malaria Information System (MIS), and sent to the CNM that appends the comprehensive, country-wide database [26].

## Results

### Participant enrolment

**Fig. 2:**
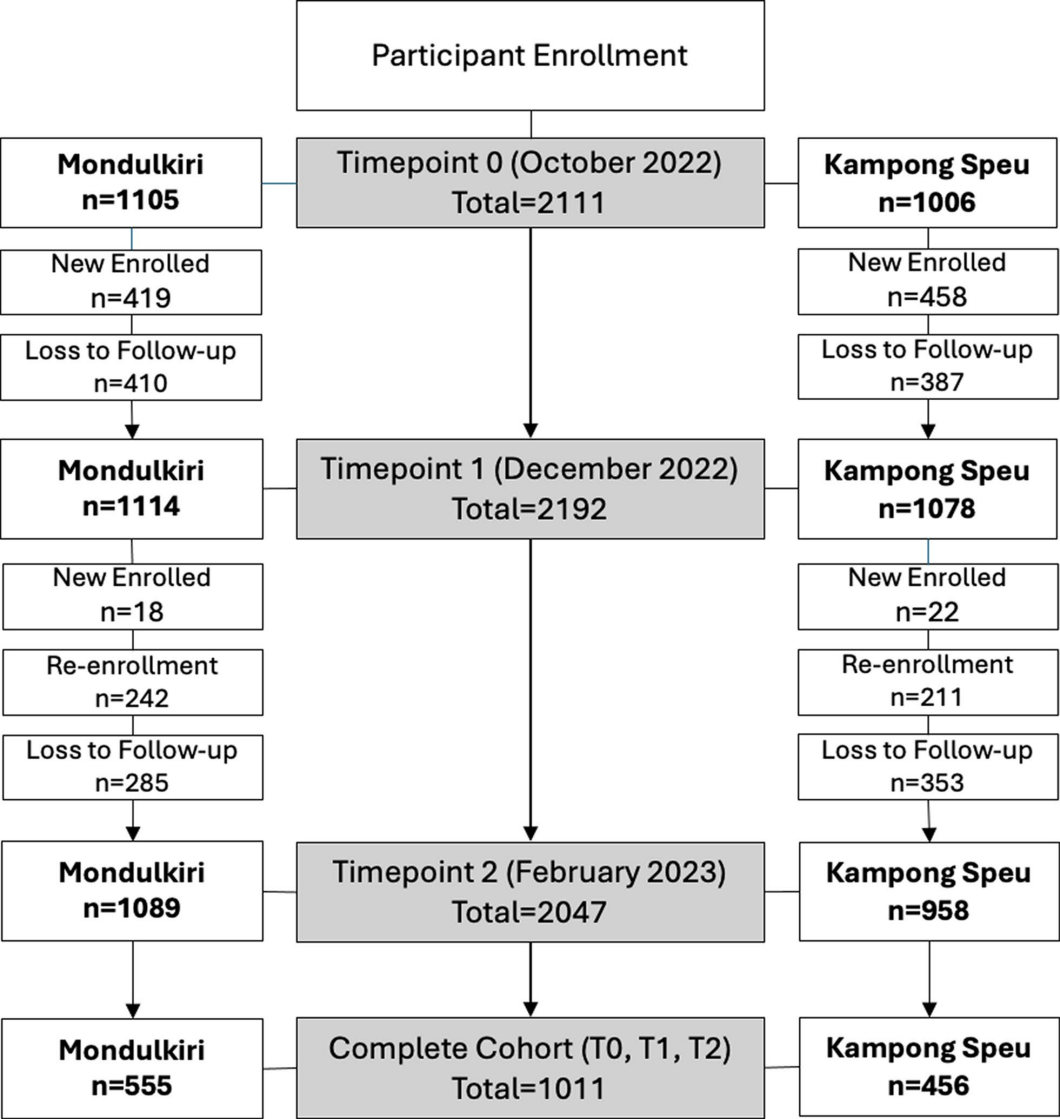
Participant Enrollment

Participant enrolment in the malaria cohort study occurred at three different time points (T0, T1, T2). In T0, a total of 2111 individuals were enrolled in the study. During T1, a total of 2192 individuals took part in the study. Out of these, 1315 were participants who continued from T0, while 877 were newly enrolled in T1. Approximately 796 participants were lost to follow-up. For T2, a total of 2047 individuals took part in the study. Out of these, 453 participants continued from T0, 40 new participants joined, and unfortunately, 638 participants were unable to be followed up with. In addition, a total of 1011 participants were enrolled at all three time points (T0, T1, and T2) (Fig. 2). Overall, 3028 people were tested with a follow-up cohort of 1011 people present at all 3 timepoints.

### Characteristics of the study population

Across all three time points of the study, there were 1,011 participants who were sampled at each point, of which 555 (54.9%) were from the province of Kampong Speu and 456 (45.1%) were from Mondulkiri. The participants were classified into three distinct groups: forest dwellers (n = 685, 67.7%), forest goers (n = 299, 29.6%), and forest rangers (n = 27, 2.7%). Participant ages ranged from 4 to 81 years with a mean age of 33.3 years (SD 15.9). The proportion of participant gender was approximately equal (55.5% females, 44.1% males). One-third of participants belonged to the indigenous minority Bunong group (32.0%), 66.8% were Khmer, and 1.2% belonged to other ethnic groups. Approximately 43.2% (n = 437) of the participants, stated that they had never been diagnosed with malaria. On the other hand, 15.8% (n = 160) of participants, had received a diagnosis of malaria at least once in their life. In addition, 40.7% (n = 411) had experienced multiple diagnoses of malaria, while a very small percentage (0.3%, n = 3) were not aware of their previous diagnosis. Of the three groups of participants, forest rangers reported the highest proportion that had been diagnosed with malaria at least once (70.37%). The main sources of family income in the last year were farming among the dweller group and the forest goer group, and for the ranger group, 63.89% of forest ranger jobs were the main source of family income. Within the forest dweller group, 94.60% of participants lived in the forest, while 55.56% of the forest ranger group lived there. Most forest goers reside more than 2 km from the forest (55.52%). The findings also suggest that participants are more likely to take precautions against mosquito bites at night, both inside and outside the house. Descriptive demographic details of the study population are shown in Table 1.

**Table 1:** Study participant characteristics at T0

Variables	N = 1011		
	Forest dweller (%)	Forest goer (%)	Forest ranger (%)
No. of individuals	685 (67.75)	299 (29.57)	27 (2.67)
Province			
Mondulkiri	323 (47.15)	205 (68.56)	27 (100.00)
Kampong Speu	362 (52.85)	94 (31.44)	0 (0.00)
Age group (year)			
4–10	72 (10.51)	4 (1.34)	0 (0.00)
11–20	130 (18.98)	33 (11.04)	0 (0.00)
21–30	138 (20.15)	92 (30.77)	7 (25.93)
31–40	138 (20.15)	58 (19.40)	13 (48.15)
41–50	99 (14.45)	57 (19.06)	5 (18.52)
≥ 51	108 (15.77)	55 (18.39)	2 (7.41)
Gender			
Male	239 (34.89)	181 (60.54)	26 (96.30)
Female	446 (65.11)	114 (38.13)	1 (3.70)
Other	0 (0.00)	4 (1.34)	0 (0.00)
Ethnic group			
Khmer	433 (63.21)	218 (72.91)	24 (88.89)
Bunong	252 (36.79)	70 (23.41)	2 (7.41)
Other	0 (0.00)	11 (3.68)	1 (3.70)
Diagnosed with malaria			
Never	332 (48.47)	97 (32.44)	8 (29.63)
1 time	104 (15.18)	53 (17.73)	3 (11.11)
2–5 times	175 (25.55)	87 (29.10)	8 (29.63)
6–10 times	33 (4.82)	24 (8.03)	2 (7.41)
≥ 11 times	41 (5.99)	35 (11.71)	6 (22.22)
Don't Know	0 (0.00)	3 (1.00)	0 (0.00)
Sources of family income in past year			
Farmer	541 (56.53)	263 (48.26)	6 (16.67)
Logging	22 (2.30)	36 (6.61)	0 (0.00)
Forest collector/forager	136 (14.21)	109 (20.00)	0 (0.00)
Ranger	1 (0.10)	0 (0.00)	23 (63.89)
Day labourer	167 (17.45)	109 (20.00)	0.00
Other	90 (9.40)	28 (5.14)	7 (19.44)
Distance from house or ranger station to the forest			
I live in the forest / Ranger Station in the forest	648 (94.60)	7 (2.34)	15 (55.56)
Within 100m	2 (0.29)	35 (11.71)	1 (3.70)
Within 200m	2 (0.29)	12 (4.01)	0 (0.00)
Within 500m	3 (0.44)	16 (5.35)	0 (0.00)
Within 1km	14 (2.04)	31 (10.37)	0 (0.00)
Within 1-2km	2 (0.29)	32 (10.70)	1 (3.70)
Over 2km	14 (2.04)	166 (55.52)	10 (37.04)
Preventing daytime mosquito bites inside the house			
No	166 (24.23)	140 (46.82)	4 (14.81)
Yes	516 (75.33)	155 (51.84)	23 (85.19)
Don't Know	3 (0.44)	4 (1.34)	0 (0.00)
Preventing nighttime mosquito bites inside the house			
No	13 (1.90)	5 (1.67)	0 (0.00)
Yes	672 (98.10)	294 (98.33)	27 (100.00)
Preventing daytime mosquito bites outside the house			
No	170 (24.82)	121 (40.47)	4 (14.81)
Yes	513 (74.89)	174 (58.19)	23 (85.19)
Don't Know	2 (0.29)	4 (1.34)	0 (0.00)
Preventing nighttime mosquito bites outside the house			
No	106 (15.47)	94 (31.44)	2 (7.41)
Yes	577 (84.23)	204 (68.23)	25 (92.59)
Don't Know	2 (0.29)	1 (0.33)	0 (0.00)
Received prophylactic medicine from the government to prevent malaria			
No	581 (84.82)	274 (91.64)	22 (81.48)
Yes	104 (15.18)	25 (8.36)	5 (18.52)
Most recently receive prophylactic medicine			
Last week	20 (19.23)	4 (16.00)	0 (0.00)
Last month	37 (35.58)	6 (24.00)	0 (0.00)
Last 3 months	12 (11.54)	9 (36.00)	0 (0.00)
Last 6 months	12 (11.54)	1 (4.00)	0 (0.00)
Last year	10 (9.62)	0 (0.00)	1 (20.00)
More than one year ago	13 (12.50)	5 (20.00)	4 (80.00)

### Prevalence and incidence of *Plasmodium* species

qPCR was used to determine the prevalence of malaria across the three time points. All cases seen were asymptomatic and represent the untreated parasite reservoir. At T0, 132/2111 were positive, with 0.47% caused by *P. falciparum*, 5.73% caused by *P. vivax*, and 0.05% being mixed infections. At T1, 87/2192 among all participants tested positive, 0.46% were positive for *P. falciparum*, and 3.51% were positive for *P. vivax*. At T2, 64/2047 of all participants tested were positive, with 0.15% being *P. falciparum* positive and 2.98% being *P. vivax* positive. At T0 in Mondulkiri, 0.63% of people tested were positive for *P. falciparum*, 4.80% for *P. vivax*, and 0.09 for mixed infections. *Plasmodium falciparum* increased to 0.81% in T1, and *P. vivax* decreased to 1.97%. At T2, *P. falciparum* decreased to 0.18% and *P. vivax* decreased to 1.65%. In Kampong Speu, the overall prevalence was 7.06% in T0, 5.19% in T1, and 4.59% in T2. Regarding the target population, at T0 the prevalence rate of forest goers was 1.79%, and 5.27% was the forest dweller group. This went up with forest goers to 1.95% in T1 and went down with the forest dweller group to 3.25%. It further decreased to 1.46% for forest goers and 3.13% for forest dweller group at T2 (Table 3).

A total of 1011 participants were present at all three-time points and represented an incidence cohort. At T0, 57 (5.64%) of the cohort participants tested positive for malaria, five (0.49%) were positive with *P. falciparum* and 52 (5.14%) for *P. vivax*. At T1, 38 (3.76%) were positive for malaria, of which 0.10% (n = 1) were positive for *P. falciparum*, and 3.66% (n = 37) were positive for *P. vivax*. At T2, 33 (3.26%) of all cohort participants tested were positive and all positive samples were *P. vivax*.

With respect to the target populations, at T0 the prevalence of malaria among forest dwellers was 41 (4.06%), while forest goers had 15 (1.48%) cases and forest rangers had only one (0.10%).

The prevalence went down with forest dwellers to 2.37%, forest goers to 1.38%, and forest rangers to 0% in T1. This prevalence further decreased to 1.88% for forest dwellers and forest goers and stayed the same at 1.38% at T2 (Table 2). Across the three time points (complete cohort participant), some individuals switched between testing positive and negative for malaria incidence between the three-time points. Out of the total 1011 cohort participants, five participants are positive in all time points, 69 individuals (6.82%) changed their status from negative in T0 to positive in T1 (new infection), and 4.74% of individuals changed their status from negative in T1 to positive in T2 (Table 3).

**Table 2:** Malaria prevalence and incidence were determined molecularly across the three-time points

Group	Time Point	Total Screened	Total PCR Positive (%)	*Plasmodium species* *total positive (%)*			Target groups *total positive (%)*		
				*falciparum*	*vivax*	*Mix*	Dweller	Goer	Ranger
All Participants (prevalence)	T0	2111	132 (6.25)	10 (0.47)	121 (5.73)	1 (0.05)	87 (4.12)	43 (2.04)	2 (0.09)
	T1	2192	87 (3.97)	10 (0.46)	77 (3.51)	0 (0.00)	50 (2.28)	37 (1.69)	0 (0.00)
	T2	2047	64 (3.13)	3 (0.15)	61 (2.98)	0 (0.00)	40 (1.95)	24 (1.17)	0 (0.00)
Participants in Mondulkiri (prevalence)	T0	1105	61 (5.52)	7 (0.63)	53 (4.80)	1 (0.09)	34 (3.08)	25 (2.26)	2 (0.18)
	T1	1114	31 (2.78)	9 (0.81)	22 (1.97)	0 (0.00)	15 (1.35)	16 (1.44)	0 (0.00)
	T2	1089	20 (1.84)	2 (0.18)	18 (1.65)	0 (0.00)	10 (0.92)	10 (0.92)	0 (0.00)
Participants in Kampong Speu (prevalence)	T0	1006	71 (7.06)	3 (0.30)	68 (6.76)	0 (0.00)	53 (5.27)	18 (1.79)	0 (0.00)
	T1	1078	56 (5.19)	1 (0.09)	55 (5.10)	0 (0.00)	35 (3.25)	21 (1.95)	0 (0.00)
	T2	958	44 (4.59)	1 (0.10)	43 (4.49)	0 (0.00)	30 (3.13)	14 (1.46)	0 (0.00)
Incidence Cohort (T0,T1,T2)	T0	1011	57 (5.64)	5 (0.49)	52 (5.14)	0 (0.00)	41 (4.06)	15 (1.48)	1 (0.10)
	T1	1011	38 (3.76)	1 (0.10)	37 (3.66)	0 (0.00)	24 (2.37)	14 (1.38)	0 (0.00)
	T2	1011	33 (3.26)	0 (0.00)	33 (3.26)	0 (0.00)	19 (1.88)	14 (1.38)	0 (0.00)

**Table 3:** The changes in malaria status between three timepoints (n = 1011)

Status change	Timepoint		Total
	T0–T1	T1–T2	
Negative to Positive	69 (6.82)	48 (4.74)	117 (11.57)
Positive to Negative	114 (11.27)	71 (7.02)	185 (18.29)

### Malaria history

All participants at T0 were asked about their lifetime history of malaria. Less than half (41%) of these participants reported that they had never been diagnosed with malaria, 16% reported being diagnosed once, 36% reported between 2–10 diagnoses in their life, and 8% reported being diagnosed with malaria more than 10 times in their life, the majority (54–58%) of which were forest dwellers. The vast majority of participants (80% in Mondulkiri and 73% in Kampong Speu) had not received a positive malaria diagnosis within the past year. Participants were also asked whether they received prophylactic malaria treatment from the government over the past year. The vast majority said they did not; only 5% in Mondulkiri and 26% in Kampong Speu reported receiving prophylaxis, most of which were dwellers (80% and 73%, respectively).

For individuals who reported having at least one malaria diagnosis in their lifetime at T0, the vast majority (77%) reported having this diagnosis more than one year ago (Table 4). When comparing Mondulkiri and Kampong Speu provinces, the latter had slightly higher levels of recent diagnosis, with forest dwellers having slightly more recent self-reported diagnoses compared to forest goers or rangers.

**Table 4:** Self-reported most recent malaria diagnosis for individuals who reported having been diagnosed at least once in their life (T0)

	Total (N = 1246)	Mondulkiri (n = 660)	Kampong Speu (n = 586)	Forest Goer (n = 482)	Forest Dweller (n = 733)	Forest Ranger (n = 31)
Never^a^	3 (< 1%)	2 (< 1%)	1 (< 1%)	1 (< 1%)	2 (< 1%)	0 (0%)
Last week	16 (1%)	2 (< 1%)	14 (2%)	3 (< 1%)	13 (2%)	0 (0%)
Last month	26 (2%)	4 (< 1%)	22 (4%)	2 (< 1%)	24 (3%)	0 (0%)
Last 3 months	54 (4%)	19 (3%)	35 (6%)	24 (5%)	29 (4%)	1 (3%)
Last 6 months	60 (5%)	22 (3%)	38 (7%)	14 (3%)	44 (6%)	2 (7%)
Last year	128 (10%)	83 (13%)	45 (8%)	51 (11%)	76 (10%)	1 (3%)
More than one year	959 (77%)	528 (80%)	431 (73%)	387 (80%)	545 (74%)	27 (87%)

^a^Never: Of the participants who had reported receiving at least one malaria diagnosis in their lifetime, three individuals then responded during the follow up question (“When was your most recent malaria diagnosis?”) that they had never been diagnosed with malaria

### Health facility-based data

Passively reported malaria case data was extracted from routine MIS from health centres in Mondulkiri (5HCs) and Kampong Speu (1HC) province, overlapping the period of the active malaria survey. From October 2022 to February 2023, a total of 13,263 cases from Mondulkiri were screened for malaria, of which 144 (1.09%) were RDT positive—41 (0.31%) were *P. falciparum* infections, and 103 (0.78%) were *P. vivax*. In Kampong Speu, a total of 4799 cases were screened for malaria, of which 10 (0.21%) were RDT positive—one (0.02%) was a *P. falciparum* infection, while nine (0.19%) were *P. vivax* (Table 5).

**Table 5:** Malaria cases, based on RDTs, from health facility records from Mondulkiri and Kampong Speu during the cohort study period (Oct2022–Feb2023)

Provinces	Name of Health Centre	RDTs Screening from Oct 2022-Feb 2023					
		Total Screened	Total malaria Positive (%)	Total *P. falciparum* positive (%)	Total *P.vivax* positive (%)	Total Male positive (%)	Total Female positive (%)
Mondulkiri	Sen Monorom	726	13 (1.79)	1 (0.14)	12 (1.65)	9 (1.24)	4 (0.55)
	Me Mang	4494	83 (1.85)	26 (0.58)	57 (1.27)	60 (1.34)	23 (0.51)
	Pu Chrey	866	6 (0.69)	1 (0.12)	5 (0.58)	5 (0.58)	1 (0.12)
	O Raing	2644	21 (0.79)	10 (0.38)	11 (0.42)	13 (0.49)	8 (0.30)
	Keo Seima	4533	21 (0.46)	3 (0.07)	18 (0.40)	16 (0.35)	5 (0.11)
	Total	13,263	144 (1.09)	41 (0.31)	103 (0.78)	103 (0.78)	41 (0.31)
Kampong Speu	Chambak	4799	10 (0.21)	1 (0.02)	9 (0.19)	10 (0.21)	0 (0.00)
	Total	4799	10 (0.21)	1 (0.02)	9 (0.19)	10 (0.21)	0 (0.00)

### Comparison of active and passive malaria prevalence

Active malaria screening in Mondulkiri and Kampong Speu provinces detected a higher prevalence of malaria compared to passive screening. The active screening involved testing asymptomatic individuals and comparing the results with the MIS malaria data from the same period. In Mondulkiri, active screening demonstrated a PCR-based prevalence of 5.52% at T0, 2.78% at T1, and 1.84% at T2. In contrast, the passive screening had RDT positive rates of 0.84% at T0, 1.83% at T1, and 0.36% at T2. This indicates a higher prevalence of malaria detected through active screening. In Kampong Speu, the overall prevalence in the active screening was 7.06% at T0, 5.19% at T1, and 4.59% at T2, while the passive screening had lower rates of 0.20% at T0, 0.52% at T1, and 0.10% at T2. Specifically, in Mondulkiri, the active screening showed *P. falciparum* rates of 0.63% at T0, 0.81% at T1, and 0.18% at T2. *P. vivax* rates were 4.80% at T0, 1.97% at T1, and 1.65% at T2. The passive screening had lower rates of *P. falciparum* and *P. vivax*. Active screening detected significantly more malaria cases compared to passive screening, with T0 showing the biggest difference (6.6 times higher in Mondulkiri and 35.3 times higher in Kampong Speu). Subsequent rounds (T1 and T2) also showed a clear advantage for active screening, with Mondulkiri detecting 1.5 to 5.1 times more cases and Kampong Speu detecting 9.9 to 45.9 times more cases (See Table 6).

**Table 6:** Comparison of active and passive Malaria screening in Mondulkiri and Kampong Speu Provinces

Site	Survey type	Malaria screening	October 2022-T0 (%)	December 2022-T1 (%)	February 2023-T2 (%)
Mondulkiri	Active	Total screened	1105	1114	1089
		Total PCR positive (%)	61 (5.52)	31 (2.78)	20 (1.84)
		Total *P. falciparum* positive (%)	7 (0.63)	9 (0.81)	2 (0.18)
		Total *P.vivax* positive (%)	53 (4.80)	22 (1.97)	18 (1.65)
		Total mix positive (%)	1 (0.09)	0 (0.00)	0 (0.00)
	Passive health Facility based MIS data	Total screened	2751	2947	2505
		Total RDT positive (%)	23 (0.84)	54 (1.83)	9 (0.36)
		Total *P. falciparum* positive (%)	3(0.11)	22 (0.75)	2 (0.08)
		Total *P.vivax* positive (%)	20 (0.73)	32 (1.09)	7 (0.28)
Kampong Speu	Active	Total screened	1006	1078	958
		Total PCR positive (%)	71 (7.06)	56 (5.19)	44 (4.59)
		Total *P. falciparum* positive (%)	3 (0.30)	1 (0.09)	1 (0.10)
		Total *P.vivax* positive (%)	68 (6.76)	55 (5.10)	43 (4.49)
	Passive health Facility based MIS data	Total screened	1009	963	997
		Total RDT positive (%)	2 (0.20)	5 (0.52)	1 (0.10)
		Total *P. falciparum* positive (%)	0 (0.00)	1 (0.10)	0 (0.00)
		Total *P.vivax* positive (%)	2 (0.20)	4 (0.42)	1 (0.10)

## Discussion

In this study, malaria prevalence and incidence were investigated in Mondulkiri and Kampong Speu provinces in Cambodia, across three-time points (T0, T1, T2) among high-risk populations, including forest dwellers, forest goers, and forest rangers living in, traveling to, and/or working in and around the forests who are exposed to *Anopheles* bites, to explore the reservoir of asymptomatic parasitaemia as the country accelerates towards malaria elimination. Data on asymptomatic parasitaemia among specific populations can support programmatic decision-making on appropriate malaria control measures to clear these reservoirs and include more targeted prevention interventions such as bite prevention tools and chemoprevention approaches.

The overall qPCR-based prevalence of malaria showed fluctuations within each location across all three-time points. In Mondulkiri, *P. falciparum* rates initially increased (0.63% to 0.81%) before declining at T2 (0.18%). *Plasmodium vivax* followed a similar decreasing trend (4.80% to 1.65%). Likewise, Kampong Speu displayed a gradual decrease in both *P. falciparum* (0.30% to 0.10%) and *P. vivax* (6.76% to 4.49%) across the time points. These fluctuations in prevalence may be influenced by factors such as changes in environmental conditions, vector control measures, human movement, and other contextual factors impacting malaria transmission.

Both infections in rangers were detected at the ranger station, suggesting that routine presumptive treatment for *P. falciparum* malaria at ranger stations could be helpful to remove remaining infections, and that existing methods are otherwise reasonable. For forest goers and dwellers, elimination of *P. falciparum* malaria could draw from the clustering of infections observed, all of which were low-density infections undetectable by RDT. The targeted provision of vector control tools and chemoprophylaxis [27] in locations with identified *P. falciparum* infections could accelerate elimination efforts, which should draw from MIS data heeding attention to Pu Trom and Pu Nhav villages in Mondulkiri Province, and Banteay Roka/Banteay Roka Kirisenchey (M) village in Kampong Speu Province, where *P. falciparum* infections were detected during this study.

For *P. vivax* malaria, infections were more common in Kampong Speu than Mondulkiri province, showing less clustering than *P. falciparum* malaria. Elimination of *P. vivax* malaria will be more challenging due to the need for radical clearance of latent hypnozoites, and will likely require the administration of radical cure for *P. vivax* malaria to all G6PD-normal individuals in villages where cases are detected, and could require active screening methods in locations with known malaria risk factors [28]. Chemoprophylaxis has already been used among these populations, with Result indicate that 5% of participants in Mondulkiri and 26% in Kampong Speu had received this over the past year, predominantly among forest dwellers. The further use of this strategy and its potential expansion could be impactful and acceptable for this population, although additional implementation steps would be necessary for the use of G6PD testing and the administration of radical cure given its potential to cause hemolysis in G6PD-deficient individuals.

A study conducted amongst 4,200 forest workers in the Mondulkiri province in 2018 also found within-village exposure to be a risk factor for malaria, with malaria prevalence being higher (8%) likely due to the earlier time frame [14]. In the 2018 study, all malaria infections detected were also asymptomatic and rapid diagnostic test negative; the occupations, gender, and ethnic groups present were similar to those in current study, further supporting the recommendation for the use of chemoprophylaxis and preventive tools for *P. falciparum* elimination and the administration of a radical cure to clear *P. vivax* malaria among G6PD-normal individuals.

This study supports prior molecular data which demonstrated that *P. vivax* infections predominate over *P. falciparum* in Cambodia in higher-incidence provinces [14, 29–32]. Higher proportions of asymptomatic *P. vivax* infections, which are regularly observed as relapses, can lead to higher immunity levels and, thus, overall lower individual parasite density [33, 34]. To address *P. vivax* reservoirs and relapses, a radical cure with primaquine (currently the only WHO-approved drug that clears *P. vivax* hypnozoites) is required. Administration of primaquine has significant limitations, including serious haemolysis risks in individuals with glucose‐6‐phosphate‐dehydrogenase enzyme (G6PD) deficiency [35]. There are access and delivery challenges associated with both G6PD testing and primaquine administration, including availability, non-compliance with treat regiments, and/or side effects, among others. Despite these challenges, countries across the Greater Mekong Subregion (GMS), including Cambodia, are actively working to scale up implementation of G6PD testing and primaquine use to address *P. vivax* reservoirs and accelerate progress toward malaria elimination. Cambodia has incorporated G6PD testing into the national strategy since 2021, by using the quantitative G6PD testing across the country. This new testing method allows for the safe administration of primaquine to a larger population, including both males and females *P. vivax*/mix patients.

The study demonstrated that forest dwellers had the highest prevalence in the incidence of malaria infection across all three-time points, followed by forest goers and forest rangers. This finding suggests that targeting these specific population groups may be important in malaria elimination efforts. Targeted drug-based interventions like IPTf for forest goers and potential Mass Drug Administration (MDA) or Mass Screen and Treat (MSAT) for dwellers could be crucial for reducing the reservoir of parasites and, therefore, transmission in these populations. Studies have demonstrated that MDA and or MSAT may be more effective in reducing transmission in areas nearing elimination [36]. The MDA programme in Cambodia uses 0.15 mg base/kg primaquine and was observed to reduce in *P. falciparum* gametocyte prevalence from 13% to 0.8% [37].

Compared to passive screening through routine malaria indicator surveys, active malaria screening identified higher prevalence rates and different trends for *P. falciparum* and *P. vivax*. Passive RDT data reported from health facilities during the same period (October 2022–February 2023) demonstrated a lower positivity rate (1.09%) compared to the active cohort study (ranging from 3.13% to 6.25%) with sensitive molecular diagnoses testing. This difference highlights the potential underestimation of malaria prevalence when relying solely on passive surveillance data and less sensitive RDTs, thereby also underestimating malaria in these areas. This suggests the importance of active screening in capturing asymptomatic cases and providing a more comprehensive understanding of malaria epidemiology compared to passive surveillance alone. This difference is attributed to the health system's missed asymptomatic infections and the lower diagnostic sensitivity with RDTs [22] compared to PCR. Asymptomatic and sub-patent cases, limited healthcare access, and alternative treatment-seeking behaviours may also contribute to this gap seen between the two types of data generation. This study demonstrates that a substantial number of infections may be missed by passive case detection. Here, passive detection may have miss approximately two-thirds of infections, particularly asymptomatic or sub-patent cases. This underestimation poses a challenge for accurately assessing the malaria burden, especially in high-risk populations. While passive case detection is cost-effective and essential for monitoring symptomatic cases, it has limitations due to its reliance on individuals seeking care. Conversely, active screening, though more sensitive, is resource-intensive and may not be sustainable long-term. Therefore, an integrated approach that combines both active and passive surveillance is recommended especially when considering malaria elimination. This strategy may focus on targeted active screening in high-risk areas and populations while maintaining a robust passive case detection system to ensure comprehensive monitoring and treatment access. Such an approach would enhance the accuracy of malaria burden assessments and facilitate better resource allocation for interventions. This emphasizes the importance of strengthening active case detection strategies beyond health facilities and integrating data from multiple sources for a more comprehensive understanding of the malaria burden.

This study highlights the importance of understanding the burden of both symptomatic and asymptomatic infections, differentiating *Plasmodium* species, and targeting interventions toward high-risk populations for achieving malaria elimination in Cambodia. Implementing a comprehensive strategy that addresses these factors will be crucial for reaching the goal of a malaria-free Cambodia. Targeting and treating the asymptomatic reservoir through active case detection and mass drug administration campaigns should be a priority. Continued investment in surveillance, prevention, and treatment strategies will be crucial in sustaining the progress made in the fight against malaria in Cambodia. The study's findings will help inform malaria elimination efforts in Cambodia and contribute to the development of effective strategies for controlling malaria in the region.

In conclusion, this study provides valuable insights into the prevalence and incidence of malaria in Cambodia and highlights the importance of targeting the asymptomatic reservoir and tailoring interventions to specific high-risk populations to achieve malaria elimination. Further studies and interventions targeting specific populations may be necessary to achieve the goal of malaria elimination in Cambodia.

## Data Availability

No datasets were generated or analysed during the current study.
